# Gene expression changes in tumor free tongue tissue adjacent to tongue squamous cell carcinoma

**DOI:** 10.18632/oncotarget.14288

**Published:** 2016-12-27

**Authors:** Linda Boldrup, Xiaolian Gu, Philip J. Coates, Lena Norberg-Spaak, Robin Fahraeus, Göran Laurell, Torben Wilms, Karin Nylander

**Affiliations:** ^1^ Department of Medical Biosciences/Pathology, Umeå University, SE – 901 85 Umeå, Sweden; ^2^ RECAMO, Masaryk Memorial Cancer Institute, 656 53 Brno, Czech Republic; ^3^ Department of Clinical Sciences/ENT Umeå University, SE – 901 85 Umeå, Sweden; ^4^ Institut de Génétique Moléculaire, Université Paris 7, Hôpital St. Louis, 75010 Paris, France; ^5^ Department of Surgical Sciences/ENT, Uppsala University, 752 36 Uppsala, Sweden

**Keywords:** tongue cancer, RNA expression, field cancerization

## Abstract

Due to the high frequency of loco-regional recurrences, which could be explained by changes in the field surrounding the tumor, patients with squamous cell carcinoma of head and neck show poor survival. Here we identified a total of 554 genes as dysregulated in clinically tumor free tongue tissue in patients with tongue tumors when compared to healthy control tongue tissue. Among the top dysregulated genes when comparing control and tumor free tissue were those involved in apoptosis (*CIDEC, MUC1, ZBTB16, PRNP, ECT2)*, immune response (*IFI27*) and differentiation *(KRT36*). Data suggest that these are important findings which can aid in earlier diagnosis of tumor development, a relapse or a novel squamous cell carcinoma of the tongue, in the absence of histological signs of a tumor.

## INTRODUCTION

Tongue squamous cell carcinoma (TSCC) is a sub-group of squamous cell carcinoma of the head and neck (SCCHN) the sixth most common cancer in the world. SCCHN is often classified as one disease although the region anatomically consists of several distinct structures. For the whole group of SCCHN the overall 5-year survival has not improved significantly over the last decades [1] and still is relatively low, around 60% [2].

The poor survival rate has been ascribed to late detection, a high frequency of relapses and death due to comorbidity. Relapses can be divided into tumor recurrences when tumor cells are not successfully eliminated by treatment and second primary tumors (SPTs) caused by an independent carcinogenetic process [3]. Numerous factors have been suggested as predictive of recurrence such as tumor stage, nodal status, tumor thickness/diameter and positive surgical margins [4–6].

One reason for development of recurrence is thought to be existence of transformed cells in areas adjacent to the primary tumor. Presence of molecular changes in the area around tumors was suggested already six decades ago as field cancerization [7]. These genetic alterations related to the neoplastic process are detectable within 7 cm from the tumor [8]. The field cancerization concept has been supported by findings of histological and molecular changes in clinically normal tissue adjacent to tumors [9–13]. In its original concept, field cancerization was defined as an extended region of tissue containing a limited number of oncogenic mutations from which a clone of malignant cells arose due to additional mutations. In a recently expanded “etiologic field effect” model, it was suggested that various etiological factors and their interactions generate a field of tissue changes favoring development of cancers [14].

Due to the rapid growth of TSCC and its potential to spread to the surrounding tissue where 20 to 40% of patients already have occult metastasis at diagnosis, early detection of the tumor is of utmost importance [15, 16]. Numerous studies have been performed searching for molecular differences between TSCC and normal tissue and many genes have been found to be differentially expressed and involved in tumor development, such as matrix metalloproteinases (MMPs) and keratins [17, 18]. In keeping with studies of other cancers that aim to identify tumor-specific alterations for diagnosis, classification and prognosis, these studies on oral SCC, including our own, have compared tumor to adjacent clinically normal tissue from the same patient and have successfully identified useful tumor-associated alterations [12, 13, 19]. Clinically normal tissue in the tumor proximity as control might, however, risk masking any field effects present. Theoretically such field changes may represent a pre-neoplastic condition in which identical oncogenic events occur before overt tumor development. Alternatively, gene expression changes may represent a tissue response to either the tumor itself or to the damaging environment from which the oncogenic events arise. In either scenario, gene expression alterations in clinically normal tissue from patients with oral cancer could be useful markers for early detection of novel or relapse tumors.

Aiming at characterizing changes indicative of tumor presence in clinically tumor free tissue, we mapped and compared changes in tissue adjacent to TSCC, the corresponding TSCC and tongue samples from healthy individuals.

## RESULTS

### Multivariate data analysis of gene expression profiles of individual patients and healthy volunteers

**Figure 1:**
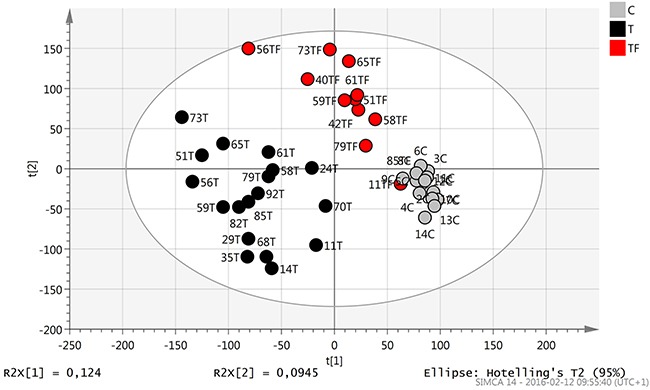
Score scatter plot (t1/t2) from PCA modelling Tumor tissue (T) samples (black dots) cluster together to the left while tumor free tissue adjacent to tumor (TF) (red dots) and healthy control tongue tissue (C) (grey dots) cluster more to the right.

A principal component analysis (PCA) model was constructed to identify outliers and clusters within the sample population (Figure 1). The PCA showed that tumor samples from TSCC patients, T (18 samples), cluster together to the left while clinically normal tumor free tongue adjacent to TSCC, TF (12 samples), and healthy control tongue tissue, C (14 samples), cluster to the right. Additionally a difference between TF and C can be observed with control samples clustering furthest away from tumors. By coloring samples according to different factors such as sex, age and RIN value separation of clusters was found to be dependent on biological differences between samples. Also the comparison of samples based on RNA extraction methods used showed no difference in expression.

### Differently expressed genes in tumor free tongue tissue adjacent tumor

**Figure 2:**
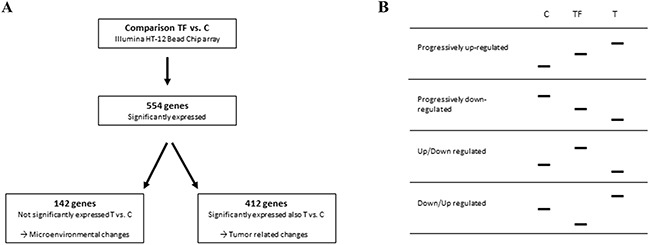
Overview of the significant gene expression analysis **A**. Comparing 12 tumor free tissue samples with 14 control samples using LIMMA statistics, p-value < 0.01 and FC > 2 showed 554 genes to be significantly differently expressed. Of these, 412 genes were also significantly differently expressed between TSCC and C, and thus called tumor related changes. The 142 genes not significantly differently expressed between T and C, represented microenvironmental changes. **B**. The expression profiles showed four different patterns, progressive up-regulation from C to TF to T, progressive down-regulation from C to TF to T, up-regulation from C to TF and down-regulation from TF to T and down-regulation from C to TF and up-regulation from TF to T.

By comparing TF (12 samples) with C (14 samples) using the illuminaHT-12 bead chip containing 47231 probes, 614 probes, representing 554 genes, were found to be differentially expressed (Figure 2A). The expression profiles showed four different patterns. Genes that were either progressively up-regulated (93) or progressively down-regulated (105) from C → TF → T, and genes showing a non-progressive pattern with either up-regulation from C → TF and down-regulation from TF → T (168), or down-regulation from C → TF and up-regulation from TF → T (188) (Figure 2B). Of the 554 genes, 142 were not significantly differently expressed when comparing T to C, and thus represented altered gene expression specific for TF. The remaining 412 genes were, on the other hand significantly differently expressed when comparing T to C based on p < 0.01, and therefore represented tumor related changes.

### Tumor free specific gene changes

Analyzing gene ontology terms for the 142 genes not significantly differently expressed between tumor and controls using WebGestalt showed cell division to be the most enriched ontology term including 10 genes, adjusted p-value 0.1542 (Table 1). By sorting the 142 genes by log fold-change (FC) between T and TF a list of the top 20 up- and down regulated genes was generated (Table 2). Of the top 20 down-regulated genes two are involved in programmed cell death, prion protein (*PRNP*) and epithelial cell transforming 2 (*ECT2*). Of the top 20 up-regulated genes three are also involved in programmed cell death, Mucin 1 (*MUC1*), Cell Death-Inducing DFFA-Like Effector C (*CIDEC*), zinc finger and BTB domain containing 16 (*ZBTB16*). qRT-PCR validation for *CIDEC* shows a good correlation with illumina expression values, Pearson´s correlation was 0.66 with p-value < 0.001.

**Table 1:** Ontology terms

Biological processes							
	Term	C	O	E	R	rawP	adjP
**TF specific**							
	Cell division	465	10	2.86	3.5	0.0006	0.1542
	Positive regulation of transcription from RNA polymerase II promoter in response to stress	5	2	0.03	65.02	0.0004	0.1542
	Chromosome segregation	146	6	0.9	6.68	0.0003	0.1542
	Regulation of attachment of spindle microtubules to kinetochore	8	2	0.05	40.64	0.0010	0.1928
	Negative regulation of transcription by competitive promoter binding	9	2	0.06	36.12	0.0013	0.2005
**Increase C-TF-T**							
	Response to type I interferon	73	11	0.36	30.62	5.17e-14	1.73e-11
	Type I interferon-mediated signalling pathway	72	11	0.35	31.04	4.41e-14	1.73e-11
	Cytokine-mediated signalling pathway	339	16	1.67	9.59	6.17e-12	1.55e-09
	Immune system process	1792	32	8.82	3.63	1.03e-11	1.73e-09
	Cellular response to cytokine stimulus	411	17	2.02	8.40	1.00e-11	1.73e-09
**Decrease C-TF-T**							
	Keratinization	44	9	0.19	47.50	1.77e-13	1.08e-10
	Epidermal cell differentiation	121	11	0.52	21.11	3.34e-12	1.02e-09
	Keratinocyte differentiation	101	10	0.43	22.99	1.47e-11	2.99e-09
	Epidermis development	455	15	1.96	7.66	6.08e-10	9.29e-08
	Epithelial cell differentiation	307	10	1.32	7.56	6.83e-07	8.35e-05

C: the number of reference genes in the category

O: the number of genes in the gene set and also in the category

E: the expected number in the category

R: ratio of enrichment

rawP: p value from hypergeometric test

adjP: p value adjusted by the multiple test adjustment

**Table 2:** Tumor free specific genes

PROBE_ID	SYMBOL	T mean	TF mean	C mean	TF vs C		T vs C		TF vs T	
		LOG values			p-value	FC	p-value	FC	p-value	FC
**Downregulated probes**										
ILMN_2228162	KRT16	12,61	10,81	12,20	8,15E-03	−1,40	4,09E-02	0,40	9,92E-05	−1,80
ILMN_2158713	IL1F9	10,00	8,32	10,73	9,13E-04	−2,41	8,14E-02	−0,73	1,37E-02	−1,68
ILMN_1676616	PTPRZ1	7,75	6,08	7,17	8,49E-03	−1,09	1,30E-02	0,58	4,32E-05	−1,67
ILMN_2091310	TMEM16A	9,82	8,17	9,17	4,04E-04	−1,01	1,98E-02	0,65	5,18E-05	−1,65
ILMN_2360415	PRNP	10,00	8,38	9,42	1,53E-04	−1,04	1,44E-02	0,58	8,12E-06	−1,62
ILMN_1717173	ECT2	8,58	6,97	8,45	9,79E-05	−1,48	5,71E-01	0,12	2,34E-05	−1,61
ILMN_1738546	LOC400578	10,82	9,26	11,28	1,48E-03	−2,02	8,45E-02	−0,45	4,33E-03	−1,56
ILMN_1659297	FZD6	8,42	6,89	7,93	5,27E-03	−1,04	4,92E-02	0,49	2,07E-04	−1,53
ILMN_2143566	SLC39A6	7,47	5,94	7,39	1,27E-04	−1,45	7,50E-01	0,08	1,14E-04	−1,53
ILMN_2167805	LUM	7,01	5,49	6,63	8,30E-05	−1,13	1,08E-01	0,39	3,55E-05	−1,52
ILMN_1797704	LOC729252	11,17	9,71	11,35	9,24E-03	−1,65	5,36E-01	−0,18	7,85E-03	−1,47
ILMN_1738558	RGS20	7,73	6,33	7,42	1,80E-03	−1,08	1,68E-01	0,31	2,63E-04	−1,40
ILMN_2063584	CLIC4	6,58	5,22	6,50	1,36E-06	−1,28	6,56E-01	0,09	4,21E-06	−1,36
ILMN_1681283	HIF1A	6,55	5,25	6,61	3,34E-04	−1,36	8,09E-01	−0,06	7,27E-04	−1,29
ILMN_1721868	KPNA2	7,10	5,83	7,11	4,06E-04	−1,28	9,70E-01	−0,01	4,61E-04	−1,27
ILMN_1750748	MGC102966	13,45	12,25	13,57	7,49E-03	−1,31	3,68E-01	−0,12	2,54E-03	−1,19
ILMN_3242120	RAP1BL	7,20	6,02	7,28	3,91E-05	−1,26	6,70E-01	−0,08	5,20E-05	−1,18
ILMN_1779852	LOC387934	11,51	10,34	11,77	6,84E-04	−1,43	1,96E-01	−0,26	2,49E-03	−1,16
ILMN_2353054	KLK5	10,28	9,11	10,44	3,59E-03	−1,33	7,31E-01	−0,17	6,69E-02	−1,16
ILMN_1760412	SHISA2	6,79	5,66	6,75	6,74E-03	−1,09	9,14E-01	0,04	1,05E-02	−1,13
**Upregulated probes**										
ILMN_2174437	CIDEC	4,66	6,86	4,91	3,96E-05	1,95	3,01E-01	−0,25	2,44E-07	2,20
ILMN_2359945	CES1	6,54	8,46	7,16	9,85E-03	1,30	1,12E-01	−0,62	4,45E-04	1,92
ILMN_1742431	LOC651309	5,70	7,62	6,18	2,20E-04	1,44	9,21E-02	−0,48	2,16E-05	1,92
ILMN_1683263	TSPAN8	5,11	6,82	5,56	9,72E-03	1,26	1,95E-01	−0,45	1,23E-03	1,71
ILMN_1684922	LOC644322	5,21	6,91	5,41	3,71E-04	1,49	4,31E-01	−0,21	4,21E-06	1,70
ILMN_2305407	ZBTB16	4,70	6,33	4,97	3,32E-03	1,36	1,95E-01	−0,28	1,35E-05	1,63
ILMN_1711422	PLEKHN1	5,62	7,17	5,71	1,13E-04	1,46	5,66E-01	−0,09	4,40E-06	1,55
ILMN_1677314	MUC1	4,83	6,33	5,14	4,28E-05	1,19	4,92E-02	−0,31	5,31E-08	1,50
ILMN_1675130	NFIC	6,82	8,32	6,58	5,89E-05	1,74	4,19E-01	0,24	1,26E-03	1,50
ILMN_1677636	COMP	6,80	8,25	6,60	2,15E-03	1,65	6,63E-01	0,20	2,32E-02	1,45
ILMN_1662587	PNPLA7	6,75	8,17	6,91	2,04E-04	1,26	4,93E-01	−0,16	6,05E-05	1,42
ILMN_1770927	KIAA1026	5,74	7,15	5,60	2,95E-05	1,56	5,01E-01	0,14	5,26E-05	1,42
ILMN_1814787	ICA1	5,66	7,06	6,06	2,33E-05	1,00	4,30E-02	−0,39	3,94E-06	1,40
ILMN_2051972	GPC3	5,32	6,68	5,54	9,24E-04	1,14	5,66E-01	−0,22	5,72E-03	1,36
ILMN_1786197	NR2F1	5,83	7,18	5,60	2,92E-05	1,58	2,75E-01	0,23	1,14E-04	1,34
ILMN_1694780	GCHFR	5,98	7,27	6,18	9,95E-04	1,09	2,02E-01	−0,20	3,52E-05	1,29
ILMN_2278653	ZNF493	6,39	7,68	6,37	1,69E-06	1,30	9,28E-01	0,02	4,21E-06	1,29
ILMN_1675258	LOC441268	5,80	7,07	5,99	4,79E-05	1,08	3,43E-01	−0,20	1,99E-05	1,28
ILMN_1670539	LOC92017	6,32	7,59	5,90	5,14E-04	1,69	9,97E-02	0,41	2,87E-03	1,28
ILMN_3244176	LOC399959	7,12	8,40	7,10	1,83E-05	1,29	9,37E-01	0,02	3,84E-04	1,27

### Progressive gene changes from control to tumor free to tumor

Of the 93 genes with progressively up-regulated expression from control to tumor free to tumor the two most enriched ontology terms were cellular response to type I interferon and type I interferon-mediated signaling pathway (Table 3). The top gene based on FC when comparing tumor free tissue and control was interferon alpha-inducible protein 27 (*IFI27*) which is involved in the type I interferon signaling pathway (Table 3).

**Table 3:** Progressively changed genes

PROBE_ID	SYMBOL	T mean	TF mean	C mean	TF vs C		T vs C	
		LOG values			p-value	log FC	p-value	log FC
**Downregulated probes**								
ILMN_1758039	KRTAP13-2	4,25	6,51	10,84	0,00018	−4,33	1,30E-23	−6,59
ILMN_2055638	KRTAP13-1	4,27	5,62	9,49	3,80E-05	−3,88	8,61E-18	−5,22
ILMN_1790252	KRT36	4,66	6,23	9,70	3,12E-04	−3,48	9,93E-22	−5,05
ILMN_1681248	TCHH	6,26	7,09	10,56	1,49E-03	−3,47	3,51E-10	−4,30
ILMN_1709708	LCE2C	5,42	6,26	9,18	8,10E-04	−2,93	3,40E-10	−3,77
ILMN_1795711	LCE2B	5,84	6,61	9,41	3,52E-03	−2,79	4,40E-08	−3,57
ILMN_1756522	LCE3A	6,82	7,91	10,67	4,58E-03	−2,76	1,27E-08	−3,85
ILMN_1653282	LCE2A	5,53	6,02	8,68	8,29E-04	−2,67	3,99E-08	−3,15
ILMN_1770228	KRT34	6,19	7,04	9,65	4,32E-03	−2,61	1,27E-07	−3,46
ILMN_1656706	LCE2D	5,45	6,17	8,71	2,49E-03	−2,53	4,08E-08	−3,25
ILMN_2045351	SPINK9	4,54	5,86	8,38	2,49E-04	−2,52	1,60E-19	−3,85
ILMN_1806059	SPRR2B	9,48	10,54	13,03	9,02E-03	−2,49	2,38E-07	−3,55
ILMN_1691410	BAMBI	7,75	7,84	10,25	8,61E-04	−2,41	1,35E-07	−2,50
ILMN_3238649	LCE6A	5,00	5,68	8,08	7,52E-04	−2,40	2,46E-09	−3,08
ILMN_1675808	TCHHL1	4,56	5,45	7,82	5,11E-03	−2,38	3,21E-10	−3,26
ILMN_1798206	KRTAP11-1	4,48	5,01	7,36	1,98E-05	−2,35	1,68E-15	−2,89
ILMN_1753439	RPTN	7,33	10,05	12,39	8,26E-03	−2,34	3,64E-09	−5,06
ILMN_1765072	KRT85	4,55	5,29	7,53	1,21E-03	−2,23	4,86E-11	−2,98
ILMN_2209088	KRTAP9-4	5,16	5,34	7,42	5,93E-04	−2,08	2,96E-06	−2,26
ILMN_1653447	PSORS1C2	5,16	5,89	7,96	1,39E-03	−2,07	2,58E-11	−2,81
**Upregulated probes**								
ILMN_2058782	IFI27	12,83	10,59	8,17	1,49E-04	2,42	1,32E-20	4,66
ILMN_2093343	PLAC8	6,18	6,12	4,51	1,89E-03	1,61	1,73E-09	1,68
ILMN_1717990	CALD1	7,49	7,26	5,67	1,56E-05	1,59	2,51E-06	1,82
ILMN_1723480	BST2	10,65	7,73	6,16	2,31E-03	1,57	7,66E-19	4,49
ILMN_1746090	STT3A	8,09	7,96	6,41	4,31E-03	1,55	3,85E-04	1,69
ILMN_1655961	C7orf54	6,72	6,52	5,00	1,39E-06	1,52	7,55E-09	1,72
ILMN_1804396	C14orf4	8,25	8,18	6,68	5,29E-03	1,49	6,38E-05	1,56
ILMN_2388547	EPSTI1	10,27	7,16	5,67	2,86E-03	1,49	6,43E-18	4,60
ILMN_2376205	LTB	8,80	6,78	5,29	4,98E-03	1,49	5,36E-17	3,51
ILMN_1736178	AEBP1	9,13	8,03	6,56	3,54E-04	1,48	2,46E-11	2,57
ILMN_1730995	AFAP1L2	9,29	8,50	7,04	2,59E-03	1,46	2,34E-08	2,25
ILMN_1784294	CPA4	8,97	8,66	7,21	1,04E-03	1,45	2,82E-06	1,76
ILMN_1760509	EOMES	6,35	5,95	4,50	2,51E-03	1,45	4,02E-07	1,85
ILMN_2143795	MGC4677	10,25	9,17	7,76	9,89E-05	1,42	1,20E-13	2,50
ILMN_1742618	XAF1	10,42	8,32	6,92	8,87E-04	1,40	1,38E-16	3,50
ILMN_1709795	RAC2	10,02	7,86	6,47	1,59E-03	1,39	1,54E-17	3,55
ILMN_1771385	GBP4	8,46	7,13	5,75	2,23E-03	1,37	5,62E-10	2,70
ILMN_1773337	DKK1	6,91	6,46	5,09	4,66E-03	1,37	1,48E-07	1,82
ILMN_1710434	TBC1D10C	6,71	6,09	4,73	1,23E-03	1,36	1,14E-08	1,98
ILMN_1745471	IRF9	10,94	9,77	8,44	1,93E-05	1,32	2,47E-16	2,49

The two most enriched ontology terms for the progressively down-regulated genes (105 genes) were keratinization and epidermal cell differentiation (Table 1), with keratin associated protein 13 (*KRTAP13*-1, *KRTAP13*-2) and keratin 36 (*KRT36*) as the top deregulated genes. The top 20 progressively up- and downregulated genes based on logFC are presented in Table 3, where mean expression values for all samples are also included. Validation of *IFI27*, *KRT36* and *KRTAP13-1* show a good correlation between illumina expression values and qRT-PCR, pearson´s correlation were 0.97;0.91 and 0.71 respectively, with p-values < 0.001 for all three genes.

### IFI27, KRT36, CIDEC, MUC1 and ZBTB16 expression in individual patients

**Figure 3:**
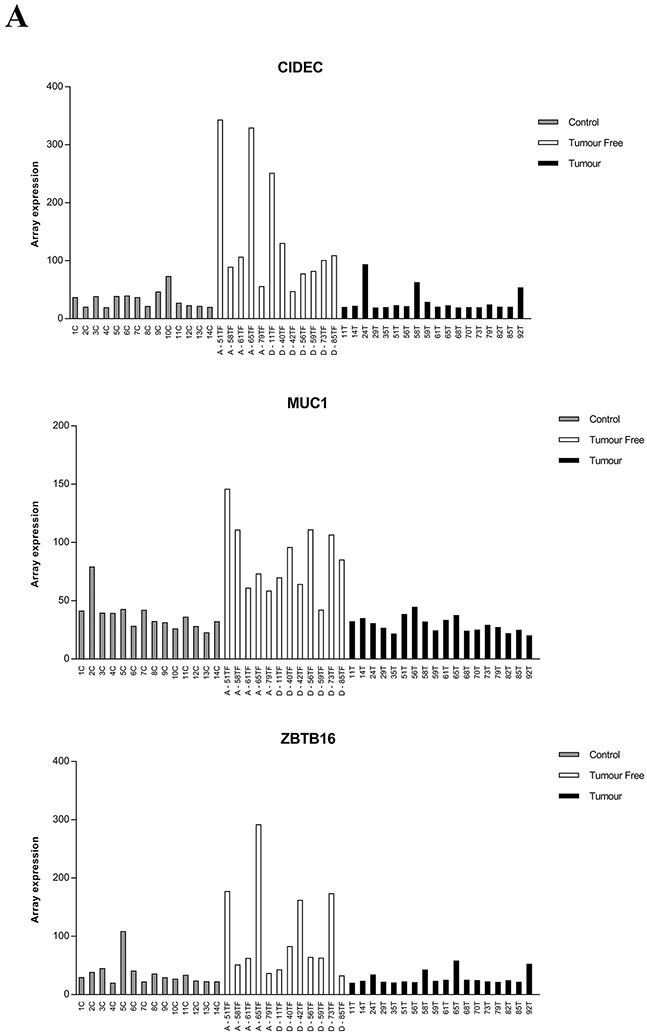

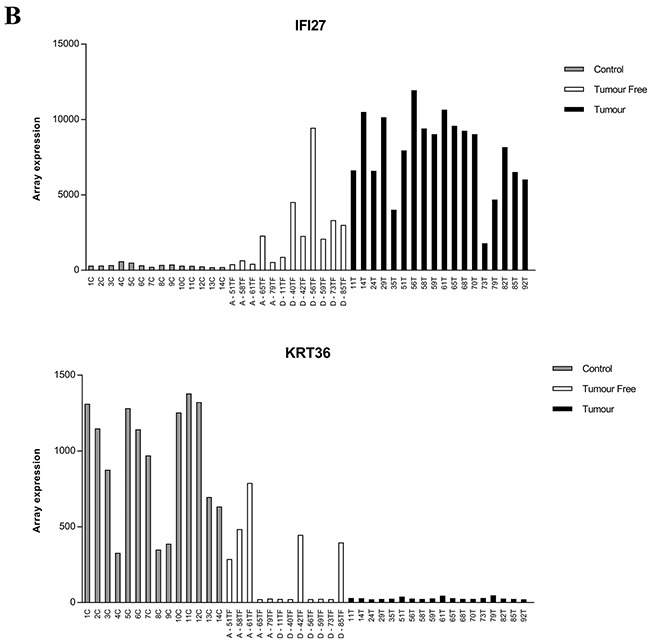
RNA expression of *CIDEC, MUC1, ZBTB16, IFI27* and *KRT36* in clinical samples The level of expression from illumina HT-12 bead chip array for each patient is plotted. **A**. Expression of *CIDEC, MUC1* and *ZBTB16* in individual patients, **B**. Expression of *IFI27*and *KRT36* expression. Control = grey bars, Tumor free = white bars, tumors = black bars. A= patient alive 5 years after diagnosis, D= patient dead 5 years after diagnosis.

Among the top genes listed, *CIDEC*, *MUC1*, *ZBTB16*, *IFI27* and *KRT36*, were selected for further analysis. Plotting RNA expression for each patient, 18 tumors (T), 12 tumor free samples (TF) and 14 controls (C) showed interindividual variations, especially in tumor free tissue (Figure 3). Comparing tumor free tissue with controls showed higher expression of *CIDEC, MUC1* and *ZBTB16* in the majority of tumor free samples.

**Figure 4:**
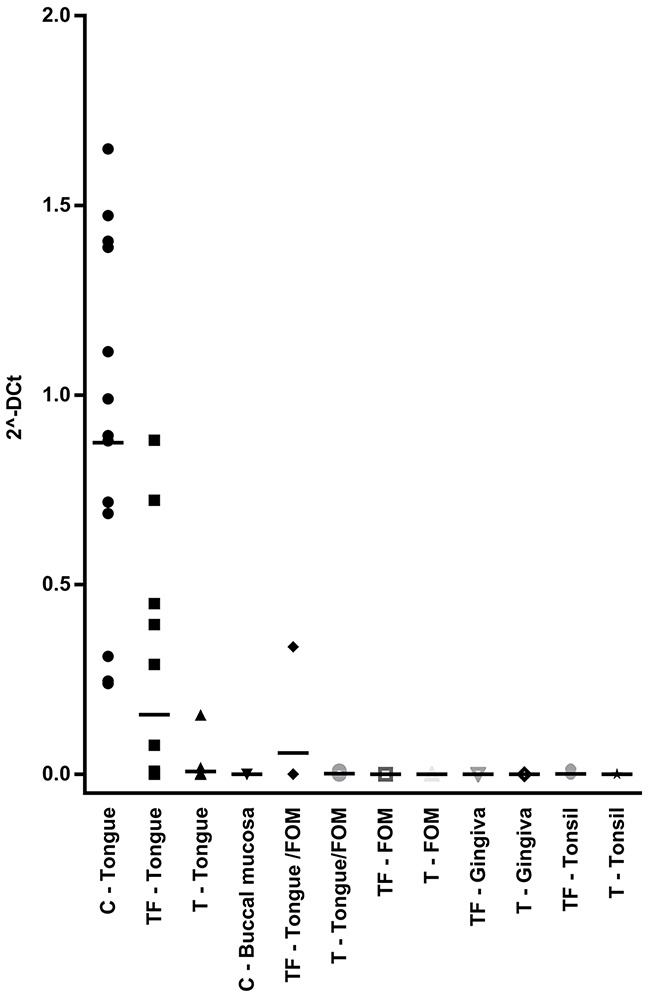
*KRT36* expression in different oral locations Levels from qRT-PCR measurement of *KRT36* in different locations in the oral cavity is shown in the dot plot. Each dot represents one patient. All locations except control tongue tissue and tumor free tongue tissue showed very low levels of *KRT36* expression. Black line indicates mean expression value for each location.

Four of the 5 patients that were still alive after 5 years had lower levels of *IFI27* in tumor free tissue compared to the seven patients that were dead. For *KRT36*, three of five patients alive expressed high levels while five out of seven dead patients expressed low levels. As Keratin 36 is not previously known as an oral keratin, its expression was evaluated in SCC, TF and C of different locations within the oral cavity using PCR. Results showed *KRT36* to be tongue specific and highly expressed in normal tongue but not in normal buccal mucosa or tumor free tissue from floor of the mouth, gingiva and tonsil (Figure 4).

## DISCUSSION

Previous studies suggested that gene expression in histologically normal oral mucosa might be useful in predicting outcome and recurrence of OSCC [13, 20]. While these studies included tissue collected from oral mucosa from different sites, tongue, gingiva and floor of the mouth [9–11] we here analyzed tissue from mobile tongue only.

Our genome-wide expression profiling revealed dysregulation of 554 genes in clinically tumor-free tongue compared to control tongue from healthy individuals. By focusing on genes that were dysregulated in uninvolved oral mucosa compared with normal oral mucosa and also dysregulated in cancer tissue a previous study identified 71 transcripts as being important in the progression of OSCC [13]. In the present study, all changes found in clinically tumor-free tongue compared to healthy controls were included irrespective of status in tumors, as tumor free tissue not necessarily displays the same molecular features as the tumor [14]. The altered gene expression seen in tumor free tongue only and not in tumor was interpreted as microenvironmental changes in response to various exposures, whereas the progressive gene expression changes seen from controls to tumor free to tumor were classified as tumor related changes in tumor free tissue.

A total of 142 genes were significantly dysregulated in tumor free tongue compared to controls, whereas their expression in controls and tumor was similar. Of the top 20 most dysregulated genes, five are involved in regulation of apoptosis. Of the three upregulated genes, (*CIDEC*, *MUC1* and ZBTB16) *CIDEC* seems to have a pro-apoptotic role [21] while *MUC1* is anti-apoptotic [22, 23] and *ZBTB16* can both be pro- and anti-apoptotic [24, 25].

CIDEC, or Fsp27, is one of three members of the CIDE-family (cell death-inducing DFF45-like effector) which is important in regulation of energy homeostasis and also linked to development of different metabolic disorders like obesity and diabetes [26]. Looking at levels of *CIDEC* all but two of the tumor free samples showed higher levels than controls, and seven of the 12 tumor free samples had levels higher than the tumor. Whether the pro-apoptotic or the energy regulatory role was the prime activity of CIDEC cannot be judged based on the present data. *MUC1* is a well-known response gene to low nutrient and hypoxia in the microenvironment and the increased expression of *MUC1* could, apart from inhibiting apoptosis, be a protective response against the stress created in the field. Notably, MUC1 has been reported to be overexpressed in a variety of epithelial cancers, including SCCHN, and also plays a key role in cancer [27]. Overexpression of MUC1 in clinically tumor free tissue thus could play a dual role in cancer development. ZBTB16, zinc finger and BTB domain containing 16 (also known as PLZF or ZNF145), is a transcription factor involved in balancing stem cell self-renewal and differentiation and is tightly regulated in cell-type and developmental stage-specific manners [28]. Reactivation of factors involved in development is often seen in tumors, accordingly this factor could also have a dual tumor stimulatory role through stem cell renewal and/or inhibition of apoptosis.

Among the tumor related factors seen in tumor free tissue, the gene ontology terms cellular response to type I interferon and type I interferon – mediated signaling pathway including *IFI27* are seen. IFI27 is suggested to be involved in proliferation of skin keratinocytes [29] and up-regulated in breast cancer, SCC of the skin and ovarian cancer [30–32]. In ovarian cancer IFI27 is associated with patient survival where high IFI27 expression correlates with poor disease free survival [30]. A similar trend was seen in our data, where the four patients with highest *IFI27* levels died within 5 years from diagnosis while the four with lowest expression are still alive 5 years after diagnosis. Low expression of IFI27 in tumor free tongue tissue thus indicates better survival. Importantly, this correlation was only seen in tumor-free tongue tissue and not in TSCC, emphasizing the importance to evaluate also corresponding tumor free tissue.

The progressively down-regulated genes showed keratinization and epidermal cell differentiation as the most enriched ontology terms. One of the identified genes with a role in epidermal cell differentiation is *KRT36*, a type I hair keratin with an unknown role in the oral cavity. By analyzing mRNA levels in different locations in the oral cavity, we found *KRT36* to be tongue specific. Levels of *KRT36* go from very high in normal control tongue tissue to extremely low in tumors, and tumor free tissue showed a big variation in expression with patients having levels comparable to either control tongue or TSCC. So far, there is no evidence that degree of differentiation or keratinization plays a role in TSCC, but the fact that KRT36 is uniquely expressed in healthy tongue and that expression decreases and almost disappears in the majority of tumor free samples indicates that it could have a role in development of TSCC.

In summary, results show upregulation of IFI27, CIDEC, MUC1 and ZBTB16, genes important in proliferation and apoptosis respectively, in clinically normal tongue adjacent to TSCC. Furthermore, data indicate a role in tumor development for KRT36, a novel keratin in the development of TSCC.

These are important findings which can aid in earlier diagnosis of tumor development, a relapse or a novel TSCC, in the absence of histological signs of a tumor.

## MATERIALS AND METHODS

### Study design and tissue acquisition

Between May 2002 and December 2010, 20 patients undergoing treatment for TSCC consented to a tumor biopsy (T) as well as a biopsy from clinically normal tumor free tissue adjacent to the tumor (TF) for research studies. All biopsies were collected from tongue tissue and were collected before treatment of the patients. For specific location of tongue biopsies see Table 4. At the same time a biopsy for diagnostic use was taken, certifying the diagnosis squamous cell carcinoma in all cases studied. Clinically normal tumor free tissue was always taken from the opposite side and the same location as the tumor. The biopsies called tumour free tissue, were just judged clinically, and not analysed histologically before they were subjected to RNA extraction. All patients had at least 5 years follow up time. Biopsies were collected from 20 patients in total, however, in this study material from 18 T and 12 TF (10 pairs with T and corresponding TF) was used. In addition, 14 tumor free volunteers provided a biopsy from lateral border of the tongue, denominated healthy control tongue tissue (C). These biopsies were taken at the same session by an experienced ENT-surgeon. For clinical data see Table 4. Samples had been consecutively collected and several of the patients are included in previous studies with different objectives [9, 19, 33]. RNA from the biopsies thus has been extracted using two different methods, RNA only (Trizol) and RNA and protein (kit from Norgen). The project was approved by the local Ethical Committee (dnr 08-003M).

**Table 4:** Clinical information

Sample ID	Age	Gender	Group	5-year survival	TNM	Location
11	78	M	T/TF	No	T2N0M0	1
14	78	F	T	Yes	T2N1M0	1
24	64	M	T	Yes	T1N0M0	2
29	64	F	T	No	T2N0M0	1
35	24	F	T	No	T2N0M0	1
40	81	F	TF	No	T4N2bM0	3
42	68	F	TF	No	T2N0M0	2
51	74	M	T/TF	Yes	T2N0M0	2
56	41	F	T/TF	No	T2N2bM0	3
58	61	M	T/TF	Yes	T1N0M0	2
59	68	F	T/TF	No	T2N0M0	2
61	70	M	T/TF	Yes	T4aN0M0	3
65	81	F	T/TF	Yes	T2N0M0	3
68	62	M	T	No	T2N0M0	2
70	71	M	T	Yes	T1N0M0	1
73	81	M	T/TF	No	T4aN0M0	3
79	61	M	T/TF	Yes	T1N0M0	1
82	19	F	T	No	T4N0M0	2
85	87	F	T/TF	No	T2N0M0	2
92	63	F	T	No	T2N0M0	1
NT1	32	F	C			1
NT2	49	F	C			1
NT5	27	M	C			1
NT3	25	F	C			1
NT4	30	M	C			1
NT6	42	F	C			1
NT7	32	F	C			1
NT8	41	F	C			1
NT9	35	F	C			1
NT10	57	M	C			1
NT11	45	M	C			1
NT12	37	M	C			1
NT13	48	F	C			1
NT14	59	F	C			1

1= Lateral border of the tongue

2= Tongue

3= Tongue with overgrowth to floor of mouth

### RNA extraction

The fresh frozen biopsies were homogenized in either trizol or lysis buffer from RNA/protein purification kit (Norgen, Canada) using a precellys (Bertin Technologies, Artigus Pres Boreaux, France). After homogenization, samples were treated according to protocols provided by the supplier. For samples extracted with trizol, chloroform was added to the homogenized sample and phase separated. RNA was precipitated by adding isopropanol followed by wash in ethanol. All RNA samples were dissolved or eluted in water and quality and quantity measured using nano-drop and Agilent RNA 6000 Nano kit (Agilent 2100 Bioanalyzer, Agilent Technologies, Santa Clara, CA, USA).

### Illumina HT-12 bead chip array

After extraction and quality control 200 ng of RNA was labelled with TargetAmp™- Nano Labelling Kit for illumina Expression BeadChip (Epicenter) to produce cRNA, which was purified using Qiagen RNeasy MinElute Cleanup kit (Qiagen). Purified cRNA, 750 ng, was hybridized to illumina HumanHT-12 v4 bead chip and analyzed with an iScan system, according to the manufacturer's manuals. For raw data see http://www.ebi.ac.uk/arrayexpress/help/FAQ.html#cite, ArrayExpress accession E-MTAB-4678.

### Data pre-processing and statistics

Raw data was exported from genome studio to R, where necq normalization was performed using the BioConductor package (http://www.bioconductor.org/). After normalization MeV software and Limma statistics (http://www.tm4.org/mev.html) were used to calculate differences between groups. For significance the criteria of p < 0.01 and FC > 2 should be fulfilled. For gene ontology enrichment terms WEB-based Gene SeT AnaLysis Toolkit (WebGestalt) (http://bioinfo.vanderbilt.edu/webgestalt/) was used.

### Multivariate data analysis

Gene expression data from microarray was analyzed with an unsupervised regression method, principal component analysis, PCA [34], to provide an overview of variation in the data and detect trends and clusters in samples and variables. Data was normalized to unit variance. SIMCA 14 software (Umetrics, Umea, Sweden) was used for multivariate data analysis.

### Confirmation of array data with PCR

Real-time quantitative PCR was used to confirm array results. For cDNA synthesis, 500 ng of total RNA was used in a 20 μl reaction with RevertAid H minus first strand cDNA synthesis kit (Thermo Scientific). cDNA was diluted 5x and 2.5μl used in each reaction with a total reaction volume of 10μl. For PCR amplification of cDNA, IQ sybr green supermix (Bio-Rad) was used in combination with primers from Primerdesign Ltd (UK) for *IFI27* (Sense: CTGGGAGCAACTGGACTCTC, Anti-sense: CCTGGCATGGTTCTCTTCTCT), and from Bio-rad for *KRT36* (Assay ID: qHsaCID0023277), *KRTAP13-1* (qHsaCED0019527 and *CIDEC* (qHsaCID0022739). Reference primers used were from Primerdesign Ltd: *GAPDH, UBC* (the company does not give out sequences for these), *LAD1* (Sense: CCTCCCACCCGTCACACT, Anti-sense: CTGCTGTAGGTTCGCTGTGT) and *RPS12* (Sense: TGCTGCTGGAGGTGTAATGG, Anti-sense: GCACACAAAGATGGGCTTGG).

Cycling conditions: enzyme activation at 95°C for 3 min, denaturation at 95°C for 15s and annealing at 60°C for 60s, the process was run in 40 cycles. Pearson´s correlation was used to calculate correlation between Illumina gene expression values and the ΔCq value from qRT-PCR.
